# Measuring Users’ Receptivity Toward an Integral Intervention Model Based on mHealth Solutions for Patients With Treatment-Resistant Schizophrenia (m-RESIST): A Qualitative Study

**DOI:** 10.2196/mhealth.5716

**Published:** 2016-09-28

**Authors:** Elena Huerta-Ramos, Maria Soledad Escobar-Villegas, Katya Rubinstein, Zsolt Szabolcs Unoka, Eva Grasa, Margarita Hospedales, Erika Jääskeläinen, Elena Rubio-Abadal, Asaf Caspi, István Bitter, Jesus Berdun, Jussi Seppälä, Susana Ochoa, Kata Fazekas, M-RESIST Group, Iluminada Corripio, Judith Usall

**Affiliations:** ^1^Parc Sanitari Sant Joan de Déu, CIBERSAM G11BarcelonaSpain; ^2^Sheba Medical CenterTel AvivIsrael; ^3^Department of Psychiatry and Psychotherapy, Semmelweis UniversityBudapestHungary; ^4^Hospital de la Santa Creu i Sant Pau, CIBERSAM G21BarcelonaSpain; ^5^Fundació TicSalutBarcelonaSpain; ^6^Center for Life Course Health Research, University of OuluOuluFinland

**Keywords:** mHealth solution, treatment-resistant schizophrenia, intervention model, qualitative research, needs assessment

## Abstract

**Background:**

Despite the theoretical potential of mHealth solutions in the treatment of patients with schizophrenia, there remains a lack of technological tools in clinical practice.

**Objective:**

The aim of this study was to measure the receptivity of patients, informal carers, and clinicians to a European integral intervention model focused on patients with persistent positive symptoms: Mobile Therapeutic Attention for Patients with Treatment-Resistant Schizophrenia (m-RESIST).

**Methods:**

Before defining the system requirements, a qualitative study of the needs of outpatients with treatment-resistant schizophrenia was carried out in Spain, Israel, and Hungary. We analyzed the opinions of patients, informal carers, and clinicians concerning the services originally intended to be part of the solution. A total of 9 focus groups (72 people) and 35 individual interviews were carried out in the 3 countries, using discourse analysis as the framework.

**Results:**

A webpage and an online forum were perceived as suitable to get both reliable information on the disease and support. Data transmission by a smart watch (monitoring), Web-based visits, and instant messages (clinical treatment) were valued as ways to improve contact with clinicians. Alerts were appreciated as reminders of daily tasks and appointments. Avoiding stressful situations for outpatients, promoting an active role in the management of the disease, and maintaining human contact with clinicians were the main suggestions provided for improving the effectiveness of the solution.

**Conclusions:**

Positive receptivity toward m-RESIST services is related to its usefulness in meeting user needs, its capacity to empower them, and the possibility of maintaining human contact.

## Introduction

In the European Union, approximately 5 million people (0.2%-2.6%) suffer from psychotic disorders [1]. Patients with schizophrenia make up the largest subgroup, and between 30% and 50% of them can be considered resistant to treatment [2,3]. Standard intervention focused on patients with treatment-resistant schizophrenia is complex because of the presence of persistent positive symptoms, extensive periods of hospital care, and increased risk of multimorbidity. The full scope of this scenario generates a high degree of suffering for patients and their families and a great financial burden on the health care system [4-6].

Study of these patients' needs is necessary for a better understanding of their psychosocial functioning, in order to develop rehabilitation goals as well as to provide them with better care [7,8]. According to the published literature, decreasing psychological distress and psychotic symptoms, keeping company and social activity, daytime activities, and information concerning the condition and treatment are the most common needs identified by patients [9-11]. This assortment of needs is similar to what caregivers and clinicians report [12-15], with nuances across countries, given the different design and functioning of the health care systems [14,16,17]. Moreover, members of professional organizations have proposed, as an important issue in the clinical intervention in treatment-resistant schizophrenia, the development and implementation of programs to promote the use of individualized treatment, taking into account the patient’s wishes and preferences whenever possible [18].

Over the last decade, different mHealth applications have been designed with the aim of meeting some of these needs. On the basis of digital technology, which offers information and therapy remotely drawing on the support of mobile devices, these solutions tend to be focused on 3 intervention areas: psychoeducation, symptom monitoring, and self-management [19-23]. Web-based psychoeducational interventions have been proven to offer patients information about the illness as well as training through systematic education programs [24]. Monitoring of the disease in mHealth interventions has usually been implemented using the Internet and mobile phone apps. It is particularly helpful in increasing medication adherence, offering medical information about patients' conditions, monitoring symptoms, and conducting cognitive behavioral therapy [25,26].

Despite the proven efficacy of these applications, we still face a lack of technological intervention models in the treatment of patients with psychotic illnesses. According to Ben-Zeev [27], Ben-Zeev et al [28], Granholm et al [19], and Rotondi et al [20], this is explained by the existence of a professional discourse on telemonitoring, with concerns about both the control that the usage of technological solutions can generate in patients and the lack of ability and willingness of patients to engage in mobile interventions. These conceptions derive from the fact that, to date, mHealth interventions for people with schizophrenia have focused on alternative means of delivering preexisting services, such as therapy, and attempting to increase adherence to medications and symptom monitoring. Thus, their potential contribution to social networks and self-management support for people with a diagnosed, serious mental illness has been overlooked [26].

With the intention of improving the quality of care of outpatients with treatment-resistant schizophrenia, an mHealth solution termed Mobile Therapeutic Attention for Patients with Treatment-Resistant Schizophrenia (m-RESIST) is being designed in the European Union. This innovative project focuses on patients with persistent positive symptoms, with the aim of empowering them, personalizing their treatment by integrating pharmacological and psychosocial approaches, and developing knowledge of the illness using predictive models to analyze historical and real-time data based on environmental factors and treatment outcomes. In the course of the project, designed by a consortium of 12 entities from the public and private sectors (including clinical and technological institutions), a system based on computer and mobile application as well as wearable devices will be developed. The system will serve patients, caregivers, and clinicians and will include the following functions: monitoring, medical-psychological assessment, intervention, and information. Contrary to previous applications focusing only on one area (education, monitoring, or self-management), m-RESIST is an integral intervention model that covers all of these features.

The objective of this study was to identify the needs and preferences of outpatients with treatment-resistant schizophrenia, informal carers, and clinicians that could be met through mHealth interventions and particularly the m-RESIST solution.

## Methods

### Design

In order to involve users in the design of the system, a qualitative study of requirements of outpatients with treatment-resistant schizophrenia was performed before the implementation of the solution. The study was carried out by 3 of the member institutions of the m-RESIST consortium: Parc Sanitari Sant Joan de Déu (Spain), Semmelweis University (Hungary), and Gertner Institute (Israel), from March to June 2015. The selection of core ideas provided below satisfies 2 requirements: to appear in the 3 countries and to be the most commonly mentioned by participants in focus groups.

### Study Participants

Outpatients with treatment-resistant schizophrenia, informal carers (relatives), and clinicians were included in the sample. There were a total of 9 focus groups, 3 in each pilot country. Each group was composed of one of the participant profiles. Additionally, 35 individual interviews were performed, which were unevenly distributed throughout the 3 institutions (Table 1). The aim of the interviews was to examine issues that were not sufficiently discussed in the focus groups, so their maximum number depended on reaching the theoretical saturation point.

Selection of participants was carried out using nonrandom sampling. All participants had to be fluent speakers of the main language in each country (Spanish, Hebrew, and Hungarian) in order to ensure that the range of needs and opinions was recorded accurately. Patients and informal carers were referred to the research teams by their treating psychiatrist, according to specific inclusion criteria described below. Once patients agreed to be part of the study, their contact numbers were provided to the research staff to arrange the date of the focus group or interview. Clinicians from the hospital and mental health center network staff were directly contacted by the research team. Before the start of the focus groups and interviews all participants were asked to provide written informed consent, after the nature of the study was fully explained to them.

Inclusion criteria, both common to all participants and specific for each profile, were established (Textbox 1).

**Table 1:** Number of people included in focus groups and interviews.

Category		Sex	Spain, n	Israel, n	Hungary, n
**Focus groups**					
	Patients	Males	7	1	2
		Females	3	5	3
	Informal carers	Males	0	2	6
		Females	5	7	4
	Clinicians	Males	4	2	3
		Females	8	7	3
	Total		27	24	21
**Interviews**					
	Patients	Males	0	10	2
		Females	4	10	5
	Informal carers	Males	4	0	0
		Females	0	0	0
	Total		8	20	7

**Common and specific inclusion criteria.**

Inclusion criteria common to all participants:

- Older than 18 years
- Basic knowledge and use of information and communications technology

Specific inclusion criteria for clinicians:

- Psychiatrists, psychologists, social workers, nurses, and case managers
- A minimum of 5 years of experience in the treatment of patients with treatment-resistant schizophrenia

Specific inclusion criteria for informal carers:

- Family members: parents, partners, and siblings
- Primary responsibility for patient’s care

Specific inclusion criteria for outpatients:

- Younger than 45 years
- Schizophrenia diagnosed according to the *Diagnostic and Statistical Manual of Mental Disorders* (Fourth Edition, Text Revision)
- A maximum of 15 years of illness progression
- More than 6 months since the diagnosis of treatment resistance
- Positive symptoms ≥4 (at least moderately ill), according to Clinical Global Impressions Scale [29]

Treatment-resistant schizophrenia is defined when adequate antipsychotic treatment is prescribed but there is no response to it (not satisfying the Andreasen remission criteria based on the Positive and Negative Syndrome Scale, PANSS) [30]:

1. Primary resistant patients defined as patients with persistent auditory hallucinations and/or delusions that have not responded to treatment with 3 adequate regimens of antipsychotic medication (including one failed trial of clozapine) for at least one year from the screening visit [31].

2. Patients with persistent psychotic symptoms (“Pseudoresistant”) owing to the following causes: low adherence, drug abuse, poor insight, isolation tendency, low involvement of caregivers in the therapeutic process, or ineligibility for treatment programs [32].

Patient eligibility for treatment-resistant criteria was confirmed following clinical evaluation using PANSS interview by trained, experienced clinicians.

Two exclusion criteria were defined regarding patients: intellectual disability and being admitted in an acute care unit.

Criteria concerning patients, as well as those related to informal carers and clinicians, were confirmed through a short questionnaire administered at the beginning of the focus groups and interviews. All participants had to consent to audiotaping of the sessions and be willing to clarify portions of the transcripts if necessary.

This study complied with the provisions of the Declaration of Helsinki [33] and was approved by the ethical committees of the 3 institutions involved.

### Procedures

Both focus groups and interviews were carried out in a venue (meeting rooms) outside the hospital areas where patients were usually treated. The average duration of the sessions was 90 minutes for focus groups and 1 hour for interviews. The participants were asked about a range of different issues, which were divided into 3 parts:

1. Outpatients' needs in their everyday life, identified by them, the informal carers, and the clinicians.

2. The role played by the health care system, focusing on their strengths and deficiencies, in order to meet these needs.

3. Opinions about the solutions originally intended to be part of the m-RESIST: online visits, alerts, data transmission system, instant messages, and a webpage specialized in treatment-resistant schizophrenia.

### Research Team

Two professionals were required to conduct focus groups: a moderator and a coordinator. The research team designed an in-depth procedure for focus groups and interviews (internal document) where we specified the question and the topics to be covered. The moderators, who proposed the different issues to talk about and guided the conversation, were professionals with more than 5 years of experience in qualitative techniques: a psychologist in the case of Spain, a sociologist in Hungary, and an art therapist in Israel. The coordinator was a sociologist in the focus groups carried out in Spain, a psychologist and a psychiatrist (depending on the group) in Hungary, and a transcriber in Israel. Coordinators were responsible for welcoming participants, taking notes, recording the conversation, and distributing the questionnaires among the participants. In the specific case of Israel, a third profile was additionally included, a psychologist, to clarify those questions related to the treatment and symptoms that emerged during the conversation. In addition, all of these professionals acted as interviewers.

There was no preexisting relationship between the research team and the group of patients and informal carers who participated in the study. In the specific case of clinicians, the research team members were acquainted with some of them by sight, although they were not colleagues.

### Theoretical Framework

The study was based on the principle of discourse analysis. This method is predicated on the understanding that there is much more going on when people communicate than simply the transfer of information. It is not an effort to capture literal meanings; rather, it is the investigation of what language does or what individuals accomplish through language. This method concerns itself with the use of language in a running discourse, continued over a number of sentences, and involving the interaction of speaker and listener in a specific situational context: mainly interviews and focus groups. The type of linguistic material is described as “performance data” and may contain features such as hesitations, clichés, and nonstandard forms such as colloquial expressions [34]. The consideration of these forms of expressions is essential in examining opinions and attitudes of patients with schizophrenia, given that they tend to show a lack of verbal fluency in their speech regarding needs because of their awareness of the social stigma attached to the illness.

## Results

### Assessment of the Receptivity to the m-RESIST Services and Applications

The range of needs reported by patients with treatment-resistant schizophrenia, their carers, and the clinical staff involved in their treatment is extremely broad and includes a large variety of areas. In this study, we focused on those needs that m-RESIST aims to meet within the following intervention modules: psychoeducational, monitoring, treatment, and self-management of the disease.

#### Psychoeducational Services: Webpage and Online Forum

With regard to medical care attention, *additional information* on treatments, medication side effects, and symptoms is the main need of the patients, according to them and their informal carers. Despite the potential usefulness of the Web in meeting this need, the majority of patients and carers are skeptical about the information submitted. This is the main reason why the possibility of having a webpage specialized in treatment-resistant schizophrenia was very much appreciated:

Participant 1: *Information about what schizophrenia is, the medication you are taking...*

Participant 2: *Yes, I agree.*

Coordinator: *Sorry?*

Participant 1: *Yes, the most suitable medication for you.*

Moderator: *What else would you like to include?*

Participant 2: *The most important thing is...it has to be a secure page...because people are so confused and some people include wrong information...* [Spain; patients group]

With regard to those issues to be included in the webpage, available treatments, side effects of medication, and symptoms are the most mentioned.

Together with the need for additional information, patients and clinicians pointed out the lack of a social network that provided *company* and emotional support as one of the main patient needs. This explains the broad acceptance of an online forum aimed at promoting contact among patients. Regarding the potential participants in the forum, both outpatients and clinicians considered that it should be restricted to outpatients, through an authorization procedure, as a way of ensuring their privacy:

Participant 1: *Part of the forum needs to be private, just for patients.* [Hungary; clinicians’ group]

The patients expressed a need to protect their privacy using online “nicknames”:

Moderator: *What do you think about virtual forums for people dealing with schizophrenia...there is a certain disclosure in this.*

Participant 1: *You don’t have to write your real name...*

Moderator: *Yes, you can use nicknames if you prefer.* [Israel; patients group]

For outpatients, the forum was also perceived as a chance to feel useful helping other people with the same illness, given that those with recently diagnosed schizophrenia could be supported by long-term patients like them:

Participant 1: *I would like...for instance, to talk about the paranoias I have had...because maybe other people have had the same ones...I don't know.*

Participant 2: *Well, I think that...on the basis of the experience gained...advice is much better, when it comes from someone experienced in the same situation. You can say “Hey, keep calm, just do this or do that.” Given what the other person has experienced...* [Spain; patients group]

The need for information shown by patients, as well as their readiness for greater contact with other people with treatment-resistant schizophrenia, attests to their potential to play a more proactive role in the management of the disease.

#### Monitoring: Data Transmission

Within the field of medical care, in addition to further information on the disease, insufficient follow-up by clinicians involved in the treatment (mainly psychiatrists) was identified as a key complaint. Longer and more frequent visits were most often mentioned as being needed, by patients, clinicians, and, especially, those family members responsible for patients' care. Therefore, a lack of immediacy and of continuity of attention was identified as an important gap in the current intervention scenario for patients with persistent positive symptoms.

The implementation of data transmission service, aimed at storing patients' patterns using a smart watch sending clinicians the information recorded, could help meet this need.

The watch could really be suitable for my son, in the event that a destabilization of his heart rates is observed...My son spends the whole day at home, with his PC, watching video clips...

Spain; informal carers group

The caregivers pointed out the relevance of wearable devices such as smart watches in monitoring the patient's condition and adherence to medical regimen:

There needs to be some kind of smart watch or other device that could let me know that my son visited his doctor...or when he doesn't take the meds—so the doctor knows about it...

Israel; informal carers group

Regarding what type of data should be recorded, sleeping patterns and rhythm of activity were the most frequently mentioned, according to informal carers. For patients and clinicians, however, the recording of mental state parameters was particularly relevant in order to prevent psychotic episodes:

A worsening of symptoms can occur anywhere, anytime, even when patients are on their way somewhere. For example when they are alone...In those moments, if patients feel distressed or unstable, this device could indicate this immediately.

Hungary; clinicians group

With the intention of increasing the effectiveness of this service, the majority of patients considered that sharing data with informal carers would be useful:

Moderator: *Who would you like to share an alarm like this with?*

Participant 1: *With my mother.*

Participant 2: *With my mother as well.*

Participant 3: *With no one.*

Participant 4: *In my case...with my mother.*

Participant 5: *With my wife.* [Spain; patients group]

Despite this broad agreement concerning the idea of sharing this information with informal carers, a written approval from patients would have to be obtained by the clinical staff before their participation.

#### Clinical Treatment: Online Visits and Constraint Messages

On the basis of patients’ and informal carers’ opinions on the need to increase the *contact between patients and clinicians*, both profiles showed a positive opinion about patients' having the chance for online visits using a webcam.

With regard to their regularity, clinicians considered that online appointments and in-person visits should take place on an alternating basis. From the patients' perspective they could be especially suitable in those specific situations in which they have difficulties attending an in-person visit, such us when they fall particularly ill:

This is a great opportunity to reach the doctor, especially in times of crisis...I would love to [have online sessions]...This is something that makes the hassle of leaving home and going all the way, unnecessary...It can really help.

Israel; interview with a patient

In addition to online visits, instant messages were also presented as a way of improving patient follow-up, in this case through mobile phones. With regard to the procedure to be followed, after initial contact with patients through a message, clinicians would assess the suitability of sending instant messages as containment or, in case of a significant deterioration in a patient’s functioning, contacting the psychiatrist. This service was perceived positively by all 3 user profiles. Apart from its usefulness in improving contact between clinicians and patients, the receptivity of these messages among participants is due to their familiarity with similar online messenger applications:

Moderator: *Can you imagine using such devices/services as chat or Skype with patients?*

Participant 1: *Yes, you can communicate by short messages and e-mails as well.* [Hungary; clinicians group]

Both outpatients and clinicians spontaneously mentioned the possibility of using instant messages in a positive way and not only to prevent a worsening of symptoms:

Participant 1: *This could assist a person to manage a kind of dialog with himself...Let's say, a personal treatment plan, goals that he needs to achieve, and if he succeeds, he gets points, but this is something that he decides for himself, maybe, with a therapist.*

Participant 2: *And positive reinforcements. The issue of positive reinforcement is the best. Research also shows this, with points and similar strategies...This is fantastic!* [Israel; clinicians group]

From the professionals' point of view, these messages could benefit the patients’ self-esteem because they would feel their progress is being appreciated, which has a positive effect on the evolution of the illness.

#### Self-Management of Tasks: Alerts

Mobile alert is the solution originally intended to meet needs related to patients’ daily tasks, in addition to those concerning the management of the disease: medication intake and medical visits.

Within the domestic sphere, patients' days begin with difficulties *getting out of bed* given feelings of tiredness. *Daily hygiene* appears as the next functioning challenge for patients.

In addition to personal care, outpatients, informal carers, and clinicians identified difficulties in dealing with *medication intake*. They concluded that although the patients are fully aware of the importance of medication to prevent worsening of symptoms, they are suspicious of its use, and that this apprehension toward medication is a common occurrence among patients, so this task should be monitored by informal carers.

The major and immediate concern is what will happen if he misses his meds...we know what a crisis is, one or two days of missed meds and the consequences can be very drastic.

Israel; interview with an informal carer

Together with medication intake, most informal carers who were part of the sample reported that they were also responsible for reminding patients of the time and date of the next visit.

Patients' difficulties in dealing with daily tasks and managing the illness explain why alerts were well appreciated by the 3 groups. From the perspective of patients, the chance of sharing these alerts with their informal carers, through an additional alert, was also well received:

Participant 1: *I would like to share it with other people, in case something happens to you...*

Moderator: *Who would you like to share them with?*

Participant 1: *I don’t know. With my father, my mother...even my brother.*

Participant 2: *Yes.*

Coordinator: *And you?*

Participant 3: *My mother.*

Participant 4: *Yes, my mother as well.* [Spain; patients group]

In general, alerts were seen positively and related with different perceived needs (Table 2). The ability of the system to generate automatic reminders for medications and appointments was also pointed out by the 3 profiles.

**Table 2:** Relation between perceived needs and the m-RESIST^a^ services.

Sphere	Needs	m-RESIST service
Personal care	Getting out of bed Daily hygiene	Mobile alert
Management of the disease	Medication intake Visit reminders	Mobile alert
Health care attention	Greater follow-up	Online visit, data transmission, instant message
	Additional information	Webpage
Relational environment	Company Mutual support	Online forum

^a^m-RESIST: Mobile Therapeutic Attention for Patients with Treatment-Resistant Schizophrenia.

### Suggestions for Improvement

Despite the usefulness ascribed to the solution, several concerns were brought to light by the participants in focus groups and interviews, which were matched by proposals on how to minimize them.

#### Personalized Data Transmission Service

The main risk perceived by outpatients, informal carers, and clinicians with regard to the implementation of the m-RESIST solution was the possibility that some of the services and applications could promote a passive role in patients. These opinions focused on data transmission service and mobile alerts.

With regard to the first of these, clinicians were particularly concerned about this risk:

I think the problem is the lack of autonomy. I mean, the person has to be capable of deciding...Why does everything patients decide have to be controlled? I mean, it is irrelevant for us, as clinicians, and can even be counterproductive, to have more information than patients want to report to us.

Spain; clinicians group

Professionals suggested adapting this service to individual patient’s needs, implementing it only for those who were less capable of managing the disease on their own and more reluctant to provide information about their symptoms to clinicians. Additionally, clinicians emphasized the need for periodically checking patients' acceptance of the service over time.

#### Flexible Number of Alerts

According to clinicians, avoiding situations stressful for users is essential in preventing a worsening of symptoms. Therefore, the need to create the right number of alerts was found to be extremely important. Both outpatients and clinicians emphasized this idea, especially the case managers, given their previous expertise in using alerts.

Participant 1: *I think...in the case of patients that argue “I usually forget to take the medication” Ok, so, I can make a note of it on your mobile phone or an alarm.*

Moderator: *Do you agree?*

Participant 2: *Yes. In the following visit patients often tell you, “I deactivated the alarm because the whole day it was buzz, buzz, buzz. It drove me crazy” So, you, as a professional, have to find another sort of strategy to help patients associate the pills with their daily habits. Because, after all, an alarm is something stressful, isn't it?* [Hungary; clinicians group]

Health care professionals and outpatients pointed out that service should be targeted to specific patient profiles, aimed at those who usually forget to take medication and miss their visits with the staff. With regard to the right number of alerts, although it would depend on individual patient needs, most clinicians set a maximum of 3 a day.

#### Alternation of Online and In-Person Visits

The need to keep in-person visits with clinicians was another concern mentioned by patients, family members, and health care professionals. Thus, online visits were perceived with skepticism and fear owing to the possibility that regular visits would disappear.

I have respect for technology, but I really feel that the human contact, when you come, once a week, to see your therapist face to face, sometimes it's the only human contact that these patients have...

Israel; clinicians group

The 3 groups suggested combining online and in-person visits, as a way of maintaining the human contact between outpatients and clinicians, and promoting the mutual contact when patients were unwilling or unable to visit clinicians.

#### Differences Between Countries

We found differences in users’ needs between the 3 studied countries (Spain, Hungary, and Israel). With regard to the current health care system, everyone asked for information about the disease (negative symptoms, side effects of medication, etc), but Israel also specified the need for information on how to deal with internalized and social stigma. In terms of resources, the 3 countries required the creation of a specific website and an online forum, but with differences (whereas in Spain it was considered that there should be a moderator in the forum, Hungary accepted the existence of a forum exclusively for patients), and Israel added the idea of a “matchmaking app” for patients. Moreover, there is a general acceptance of the m-RESIST solution in Spain and Hungary, but in Israel there is more reluctance to it, considering it a supplementary treatment to the currently established treatment in any case. There are also different views on some sections: regarding the online visits, Hungarian clinicians did not agree with it, because it would increase their work; the alerts are mainly considered as reminders in Spain and Hungary, whereas Israel would extend them to customized messages; finally, regarding the smart watch device, the Spanish patients think that wearing it could be stigmatizing, whereas in Hungary there is great worry about the responsibility of maintenance and having to assume the costs in case of loss, and in the Israel group the opinion of the matter is unknown.

## Discussion

Despite the proven usefulness of mobile technology in mental health care, to date only a limited number of mHealth solutions have been implemented [28,35]. Concerns have been raised about the cognitive limitations of people with severe mental illness in dealing with Web-based and mobile applications [23,36]. The few solutions designed tend to be focused on a specific intervention area, mainly within the psychoeducational field [20] or as a way to improve adherence to medication through telemedicine systems [25,37]. Therefore, the design of an integral intervention model involving different areas of patients' attention has been left aside.

Our study provides preliminary indications that outpatients with treatment-resistant schizophrenia and positive symptoms are willing to use the services that were originally intended to be part of the m-RESIST solution and feel capable of doing so. These include online visits, instant messages, data transmission, and alerts. This broad acceptance of the solution is also supported by informal carers and clinicians. This is largely due to the capacity of m-RESIST to meet the most relevant needs in the outpatients' lives [9,12] and the possibility of encouraging them to assume a more active role in the management of their day-to-day lives [38,39]. Nevertheless, in order to further increase their suitability, it is important to consider the need to modify the initial approach of some services. In the case of data transmission service, users pointed out the importance of personalizing the service according to the capacity of patients to self-manage the illness. Mobile alerts should also be focused on those patients who usually forget to take medication or miss their medical appointments. The number and frequency of alerts have to be previously agreed upon with patients, in accordance with previous studies [19]. Regarding online visits, the importance given to the human contact in schizophrenia treatment explains users' proposal for combining them with in-person visits.

Further studies on the point of view of users toward mHealth solutions are needed [40]. It is important to consider the effects of gender and age on the patients’ perspectives when identifying and explaining the differing use of technology among patients [11,41-43]. In addition, the suspicions of clinicians regarding the possibility of using technological solutions in patient care should be taken into account in the design of future technological solutions. In the current digital era, this implies the need to prove the advantages of technology in their everyday work to them and encouraging them to actively use it.

## Abbreviations

m-RESIST: Mobile Therapeutic Attention for Patients with Treatment-Resistant Schizophrenia
PANSS: Positive and Negative Syndrome Scale
